# p70 Ribosomal Protein S6 Kinase Is a Checkpoint of Human Hepatic Stellate Cell Activation and Liver Fibrosis in Mice

**DOI:** 10.1016/j.jcmgh.2021.09.001

**Published:** 2021-09-17

**Authors:** Florian P. Reiter, Liangtao Ye, Andrea Ofner, Tobias S. Schiergens, Andreas Ziesch, Lydia Brandl, Najib Ben Khaled, Simon Hohenester, Ralf Wimmer, Renate Artmann, Yulong He, Serene M.L. Lee, Doris Mayr, Changhua Zhang, Alexander L. Gerbes, Julia Mayerle, Gerald Denk, Enrico N. De Toni

**Affiliations:** 1 Department of Medicine II, University Hospital, LMU Munich, Munich, Germany; 2 Division of Hepatology, Department of Medicine II, University Hospital Würzburg, Würzburg, Germany; 3 Liver Center, University Hospital, LMU Munich, Munich, Germany; 4 Center for Digestive Diseases, The Seventh Affiliated Hospital, Sun Yat-sen University, Shenzhen, China; 5 Department of General, Visceral and Transplantation Surgery, University Hospital, LMU Munich, Munich, Germany; 6 Biobank of the Department of General, Visceral and Transplantation Surgery, University Hospital, LMU Munich, Munich, Germany; 7 Institute of Pathology, LMU Munich, Munich, Germany; 8 Transplantation Center Munich, University Hospital, LMU Munich, Munich, Germany

**Keywords:** Fibrosis, Cirrhosis, Chronic Liver Disease, Transforming Growth Factor-β, Platelet-Derived Growth Factor BB, α-SMA, α-smooth muscle actin, CLD, chronic liver disease, GFAP, glial fibrillary acidic protein, HSC, hepatic stellate cell, pmHSC, murine hepatic stellate cell, NASH, nonalcoholic steatohepatitis, p70S6K, p70 ribosomal protein S6 kinase, PDGF, platelet-derived growth factor, phHSC, primary human hepatic stellate cell, siRNA, small interfering RNA, TGF-β, transforming growth factor-β, WST, water-soluble tetrazolium, Wt, wild-type

## Abstract

**Background & Aims:**

Progression of chronic liver disease (CLD) to liver cirrhosis and liver cancer is a major global cause of morbidity and mortality. Treatment options capable of inhibiting progression of liver fibrosis when etiological treatment of CLD is not available or fails have yet to be established. We investigated the role of serine/threonine kinase p70 ribosomal protein S6 kinase (p70S6K) as checkpoint of fibrogenesis in hepatic stellate cells (HSCs) and as target for the treatment of liver fibrosis.

**Approach & Results:**

Immunohistochemistry was used to assess p70S6K expression in liver resection specimen. Primary human or murine HSCs from wild-type or p70S6K^–/–^ mice as well as LX-2 cells were used for in vitro experiments. Specific small interfering RNA or CEP-1347 were used to silence or inhibit p70S6K and assess its functional relevance in viability, contraction and migration assays, fluorescence-activated cell sorting, and Western blot. These results were validated in vivo by a chemical model of fibrogenesis using wild-type and p70S6K^–/–^ mice*.* Expression of p70S6K was significantly increased in human cirrhotic vs noncirrhotic liver-tissue and progressively increased in vitro through activation of primary human HSCs. Conversely, p70S6K induced fibrogenic activation of HSCs in different models, including the small interfering RNA–based silencing of p70S6K in HSC lines, experiments with p70S6K^–/–^ cells, and the pharmacological inhibition of p70S6K by CEP-1347. These findings were validated in vivo as p70S6K^–/–^ mice developed significantly less fibrosis upon exposure to CCl_4_.

**Conclusions:**

We establish p70S6K as a checkpoint of fibrogenesis in vitro and in vivo and CEP-1347 as potential treatment option that can safely be used for long-term treatment.

## Graphical abstract

**Figure:**
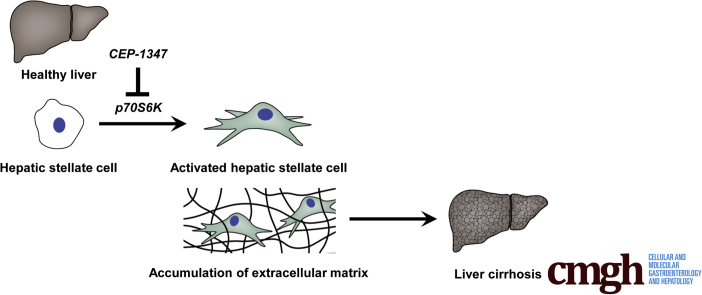

**Summary**

Targets and medical approaches to prevent progression of chronic liver disease to liver fibrosis or cirrhosis are urgently needed. We describe p70S6K being dysregulated in human liver fibrosis and define this protein as checkpoint of human hepatic stellate cell activation and liver fibrosis in vivo. Furthermore, we provide a clinical viable approach to inhibit this protein by the employment of CEP-1347.

Chronic liver disease (CLD) with its associated mortality and morbidity represents a considerable and increasing global socioeconomic problem.^1^^,^^2^ Independently of its underlying etiology, CLD can cause liver fibrosis, eventually progressing to liver cirrhosis and its complications. Once liver cirrhosis becomes clinically apparent, 5-year mortality without liver transplantation amounts to 85%^3^ owing to the clinical complication such as variceal bleeding, ascites, kidney failure, and the occurrence of hepatocellular carcinoma, currently the most common cause of death in patients with liver cirrhosis and the fourth most common cause of tumor-related death worldwide.^3^, ^4^, ^5^, ^6^, ^7^

Several advances have been made in the therapy of CLD, the latest and most remarkable perhaps being the development of direct-acting antiviral agents for the treatment of chronic hepatitis C.^8^ However, for other CLD, an effective medical treatment is still lacking, only partially effective, or effective only in a subgroup of patients.

Establishing a medical treatment capable of directly targeting fibrogenesis and delaying or abolishing the progression of fibrosis will be indispensable to reduce CLD-related morbidity and mortality until causal treatment options are established. The relevance of this issue is highlighted by the epidemiological trend of nonalcoholic steatohepatitis (NASH), a condition poorly responsive to treatment and due to become the leading cause of CLD and mortality globally.^9^^,^^10^ Unfortunately, several promising agents, such as simtuzumab or selonsertib, failed to meet their primary endpoint of inhibiting fibrosis in the treatment of CLD.^11^, ^12^, ^13^

The lack of treatment options in liver fibrosis to complement existing etiological treatment therefore represents an unmet medical need for an ever-increasing number of patients worldwide.

On the mechanistic level, liver fibrosis is thought to be caused mainly by hepatic stellate cells (HSCs) that, upon activation, transdifferentiate into myofibroblasts, cause deposition of extracellular matrix, and induce portal hypertension by contractility.^14^, ^15^, ^16^

Profibrotic activation of HSCs can, in turn, be initiated at different sites, eg, the activation of transforming growth factor-β (TGF-β) or of platelet-derived growth factor BB (PDGF-BB), 2 molecules that trigger intracellular signaling converging to activate the PI3K-AKT-mTOR signaling.^17^, ^18^, ^19^ However, attempts at blocking these receptors or the PI3K-AKT-mTOR axis by currently available agents poses several safety issues concerning long-term tolerability of their antiproliferative and immunosuppressive effects.

To establish a potentially accessible molecular target for antifibrotic treatment, we chose to investigate the serine/threonine kinase p70 ribosomal protein S6 kinase (p70S6K), a molecule phosphorylated downstream of mTOR and possibly representing a distal effector of fibrogenesis, with the rationale that its pharmacological inhibition could be safer and more tolerable than that of its upstream targets. To this regard, we also assessed the potential use of CEP-1347 as p70S6K inhibitor, an agent for which extensive clinical research has proved to be safe and very well tolerated for long-term treatment in a previous clinical phase III trial for Parkinson’s disease.^20^

## Results

### p70S6K expression is significantly increased in human liver resection specimens from cirrhotic patients compared with nonfibrotic liver tissue, and expression of p70S6K increases during the activation of primary human HSCs

**Figure 1:**
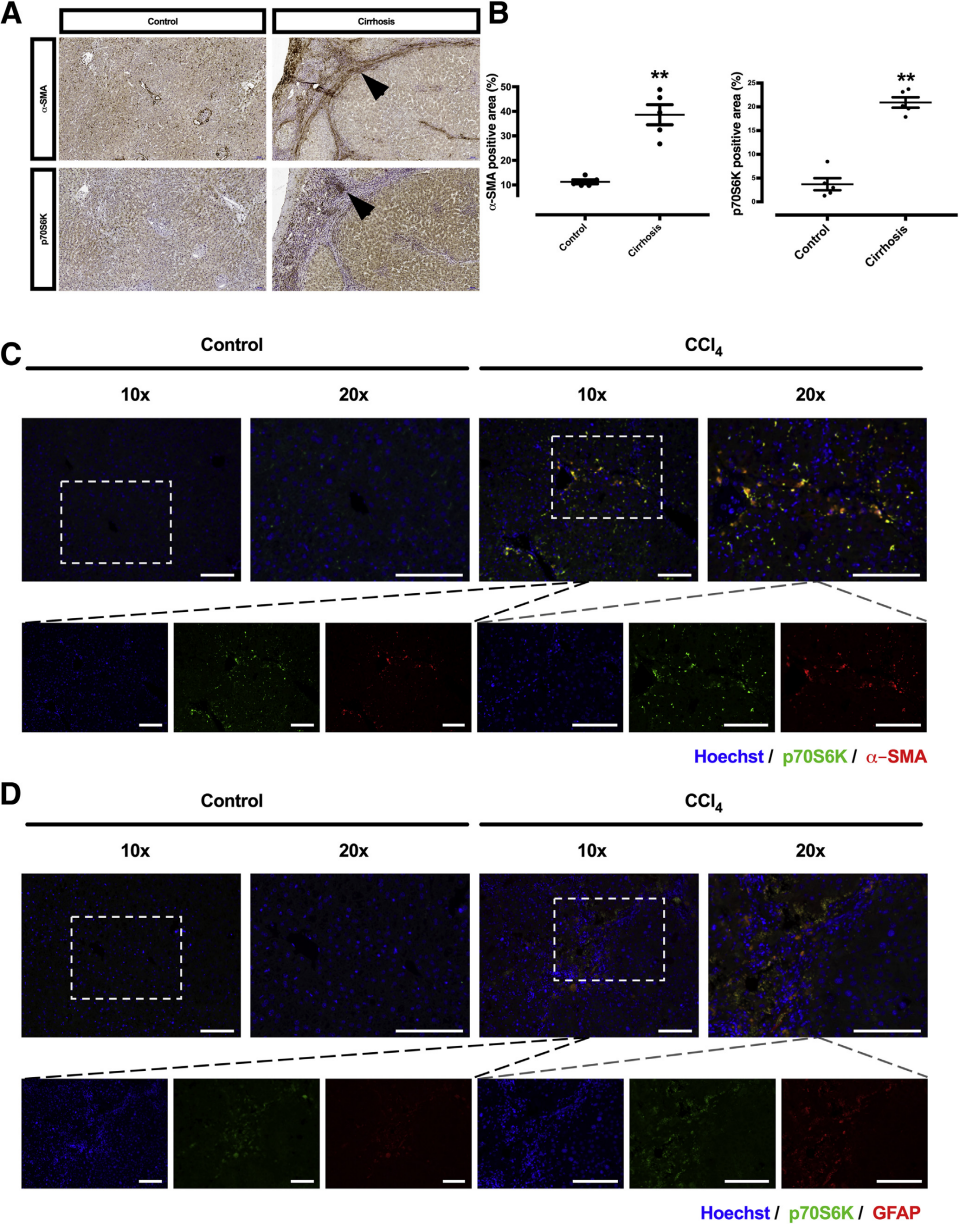
**p70S6K staining is higher in cirrhotic vs healthy livers.** (*A*) Representative images showing immunohistochemical staining of α-SMA and p70S6K in cirrhotic and healthy livers (brown staining and arrows). α-SMA staining is observed along the fibrous septa; p70S6K-positive cells are visible as clusters along or within the septa. (*B*) Immunohistochemical staining of α-SMA and p70S6K was quantified automatically from whole slide scans from the respective tissues samples. The data express the area with positive staining as a percentage of the total area (n = 5; ∗∗*P <* .01; *t* test). (*C*, *D*) A costaining of Hoechst (blue), p70S6K (green), and α-SMA or GFAP (both red) was performed in mice treated with vehicle (nonfibrotic control) or CCl_4_. Control mice show a lower expression of p70S6K vs CCl_4_-treated animals. The images show an overlapping (yellow) staining pattern of p70S6K (green) and α-SMA or GFAP (red). Scale bars = 100 μm.

**Figure 2:**
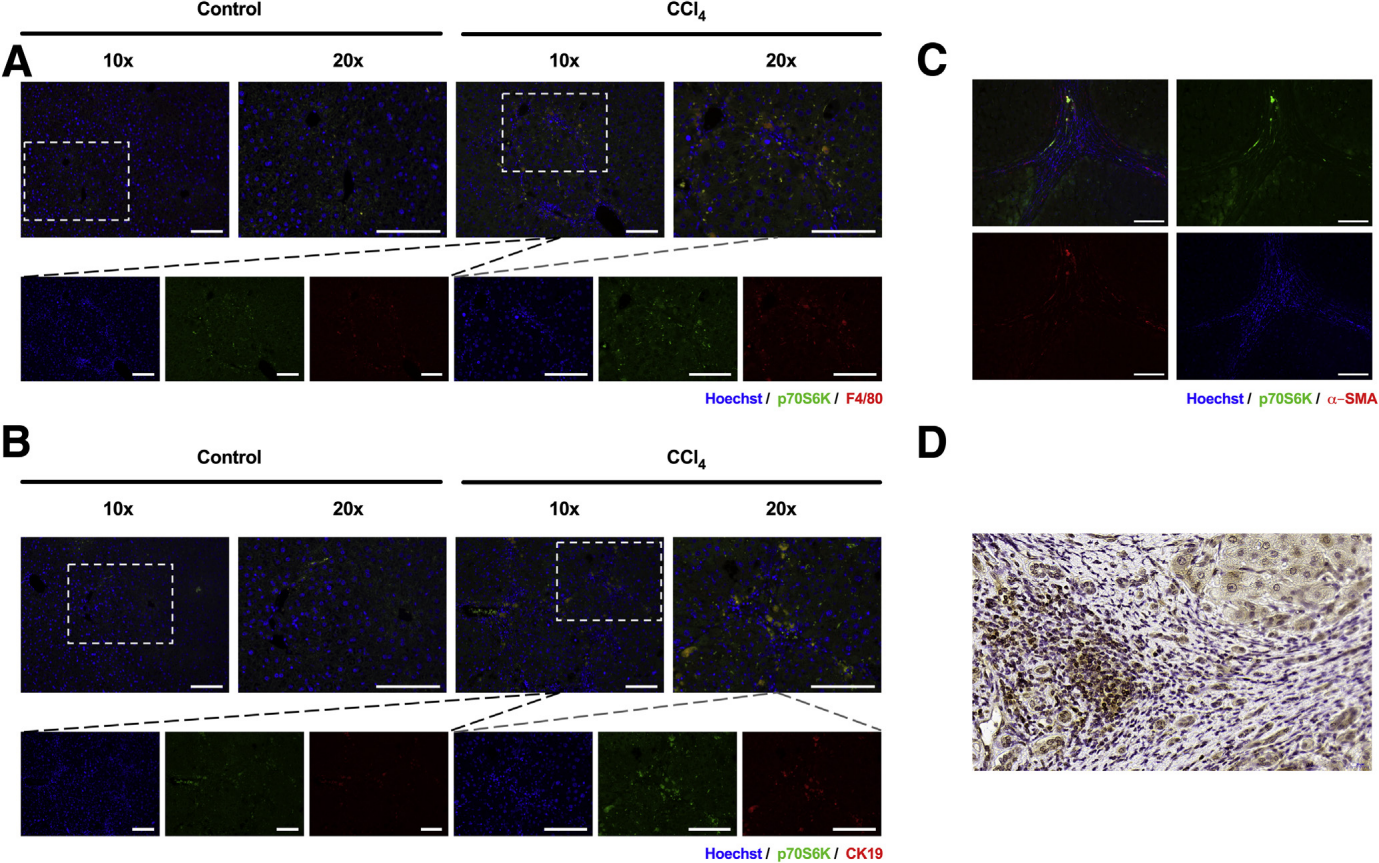
**Costaining of p70S6K and markers of nonparenchymal liver cells was done.** (*A*) Costaining of Hoechst (blue), p70S6K (green), and (*A*) F4/80 or (*B*) CK19 (red) was performed in mice treated with vehicle or CCl_4_ (scale bars = 100 μm). (*C*) Costaining of Hoechst (blue), p70S6K (green), and α-SMA (red) was performed in human liver samples from patients with liver cirrhosis. An overlapping expression of p70S6K (green) and α-SMA (red) can be observed in the septa (scale bars = 100 μm). (*D*) Nuclear staining of p70S6K in cirrhotic liver tissue (scale bar = 20 μm).

To assess whether p70S6K is differentially expressed in liver tissue with advanced stages of fibrosis or cirrhosis in comparison to tissue from healthy liver, immunohistochemical staining for p70S6K, along with immunohistochemical staining of α-smooth muscle actin (α-SMA), an established marker of HSC activation, was performed and quantified in surgical liver tissue specimens from patients with and without liver cirrhosis who had undergone partial hepatectomy. Staining for α-SMA was significantly higher in cirrhotic livers and was detectable predominantly along the fibrotic septa. Staining for p70S6K was also approximately 6-fold higher in cirrhotic liver compared with healthy liver tissue (Figure 1*A* and *B*). p70S6K was costained with α-SMA or glial fibrillary acidic protein (GFAP) in mice treated with CCl_4_ but not in vehicle-treated animals (nonfibrotic controls). This supports the observation that p70S6K is specifically increased in HSCs (Figures 1*C* and *D* and 2*C*). However, expression of p70S6K was not restricted to HSCs because p70S6K staining was observed in association with non-HSC markers such as F4/80 and cytokeratin 19 (CK19) (Figure 2*A* and B).

**Figure 3:**
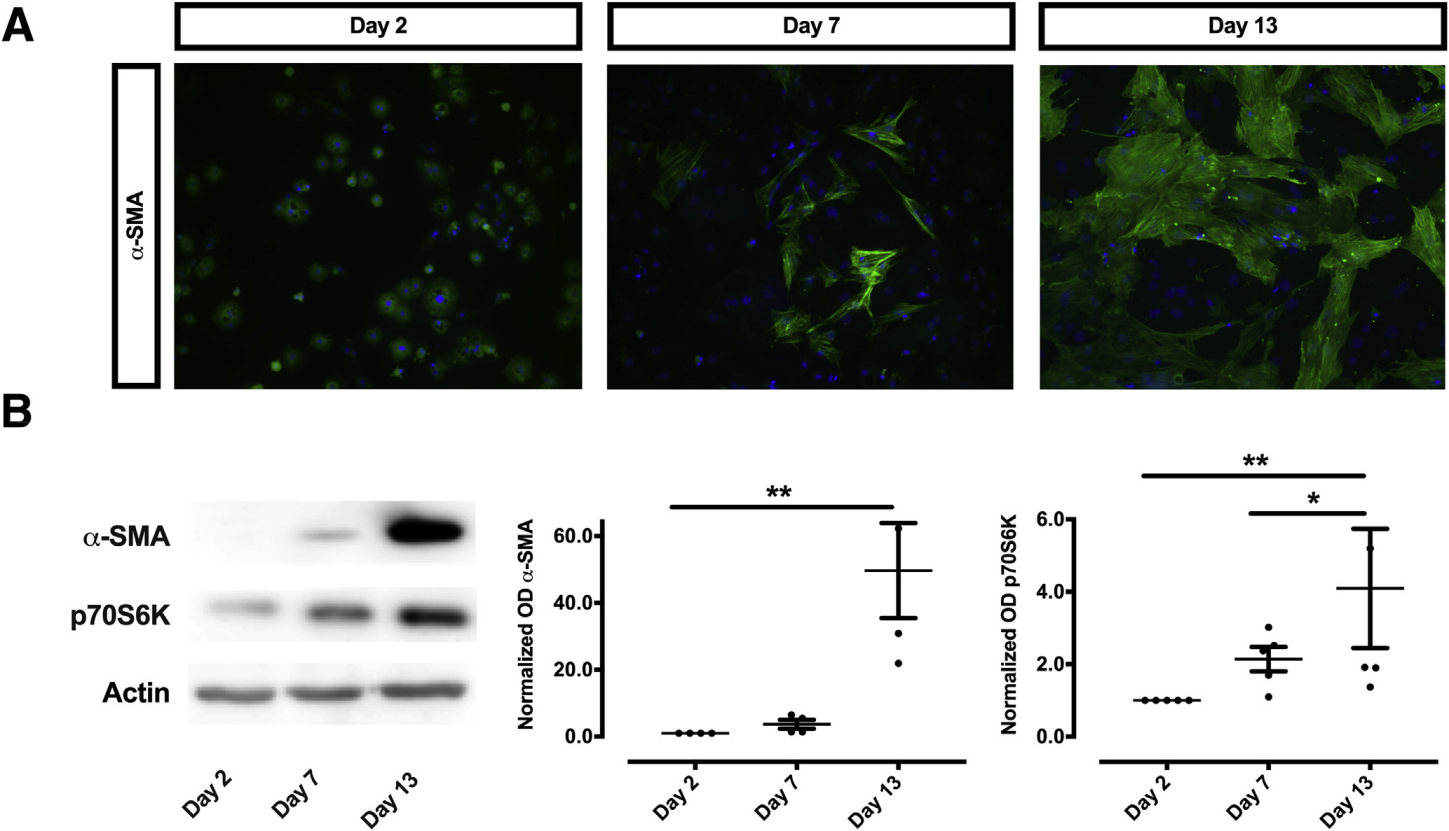
**Expression of p70S6K increases during the activation of phHSCs.** (*A*) phHSCs were seeded on uncovered plastic to cause their activation. A time-dependent, progressive activation of HSCs is evident with increasing green immunofluorescence of α-SMA, which was most prominent on day 13 after isolation. (*B*) Likewise, a progressive increase of both α-SMA and p70S6K could be seen by Western blot (left panel). Densitometry of protein-expression were quantitated from at least 4 samples and expressed as density units relative to the baseline (right panel; ∗*P <* .05; ∗∗*P <* .01; analysis of variance).

Because HSCs are thought to be the central regulators of fibrogenesis, in a next set of experiments we aimed to assess the role of p70S6K on HSC activation. For this purpose, we used a surrogate model of HSC activation by seeding primary human HSCs (phHSCs) on uncovered plastic dishes for 13 days to induce their spontaneous transdifferention to activated myofibroblasts.^21^ We observed that the expected increase of α-SMA, a hallmark of activation in these cells over 3 different time points (on days 2, 7, and 13 after isolation) was accompanied by an increased expression of p70S6K, as shown by Western blot (Figure 3). In summary, both the histological examination and the progressive increase of p70S6K during HSC activation support the hypothesis that this molecule plays a causal role in the process of fibrogenesis.

### Silencing of p70S6K counteracts TGF-β–dependent activation of LX-2 cells and spontaneous activation of isolated primary cells

**Figure 4:**
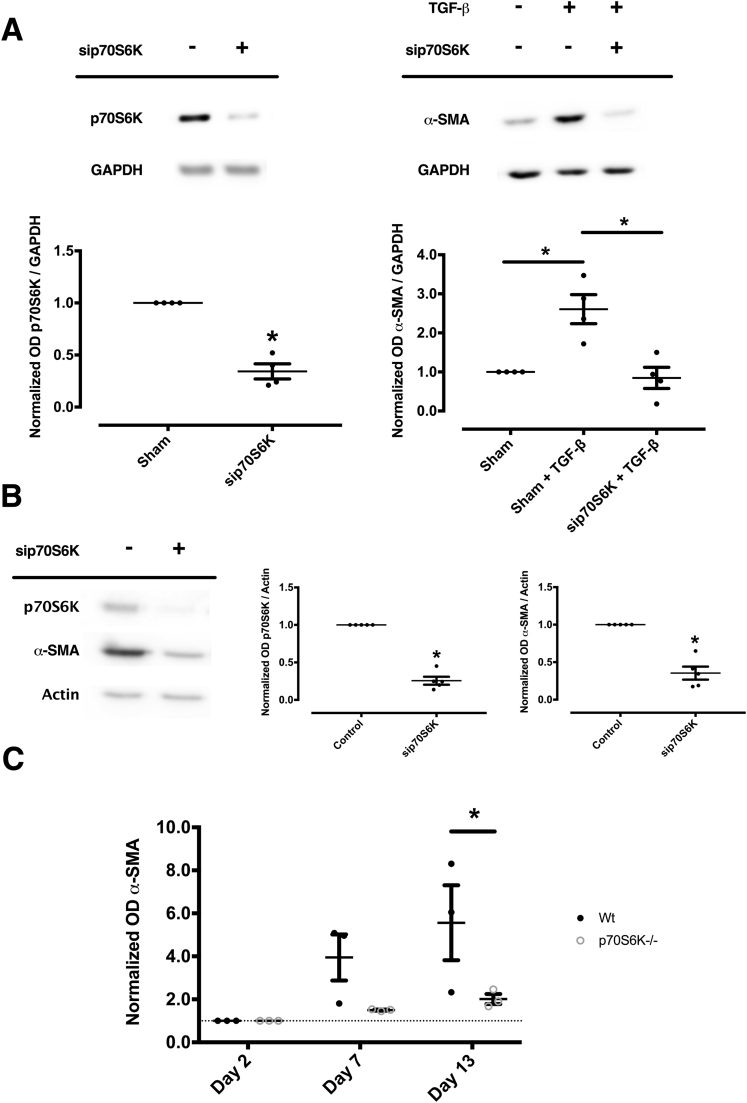
**Silencing of p70S6K abolishes TGF-β–dependent activation of human LX-2 cells and the spontaneous activation of phHSCs and pmHSCs.** (*A*) LX-2 cells were transfected with p70S6K-targeting siRNA or control siRNA (β-galactosidase–targeting siRNA). Silencing of p70S6K, confirmed by Western blot (left panel) (n = 4; ∗*P <* .05; *t* test), inhibited the activation of HSCs, as shown by the abolished increase of α-SMA caused by co-incubation with TGF-β for 24 hours (right panels) (n = 4; ∗*P <* .05; Kruskal-Wallis test). (*B*) Similar results as in *A* were obtained with phHSCs plated on uncovered plastic wells and harvested after undergoing continuous incubation with siRNA targeting p70S6K or control siRNA for 7 days. The illustrations and the quantitative assessment shown are representative of at least 4 separate experiments (∗*P <* .05; *t* test). (*C*) pmHSCs were isolated from age-matched Wt and p70S6K^–/–^ mice. Protein expression of α-SMA was measured by Western blot and expressed as fold-increase vs baseline levels on day 2. pmHSCs from p70S6K^–/–^ mice showed a smaller increase in α-SMA protein expression over time (n = 3; ∗*P <* .05; analysis of variance).

To assess whether the observed increase of p70S6K is an actual cause of fibrogenic HSC activation, p70S6K was silenced by small interfering RNA (siRNA) both in LX-2 cells and in isolated phHSCs. As shown in Figure 4*A*, silencing of p70S6K inhibited the activation of HSCs, as judged by the lack of α-SMA expression upon co-incubation with TGF-β in LX-2 cells. Similar results were obtained with phHSCs plated on uncovered plastic wells and harvested after undergoing incubation for 7 days with siRNA targeting p70S6K or control siRNA (Figure 4*B*).

**Figure 5:**
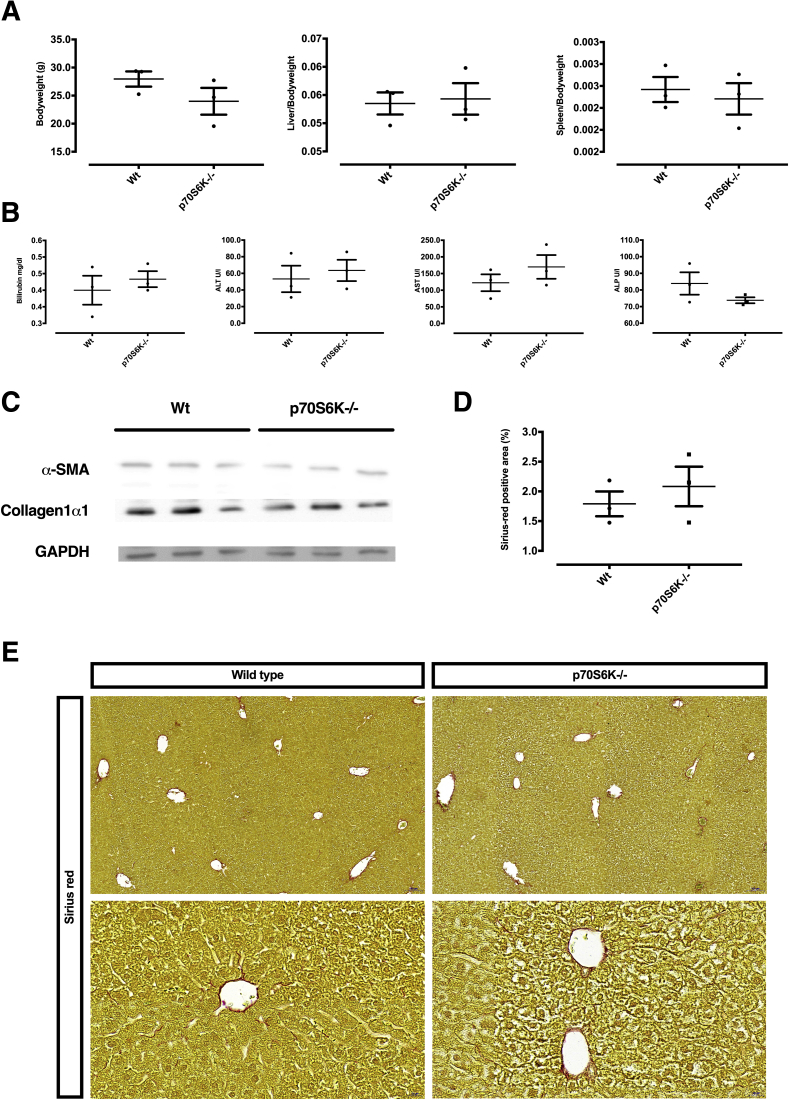
**Spontaneous phenotype of p70S6K^–/–^ mice in comparison with Wt mice.** (*A*, *B*) Comparison of age-matched Wt mice vs p70S6K^–/–^ mice did not show significant differences in body, liver, or spleen weight or in serum bilirubin, alanine aminotransferase (ALT), aspartate-aminotransferase (AST), and alkaline phosphatase (ALP). (*C*) Likewise, no differences were detected in the expression of α-SMA and collagen 1α1. (*D*, *E*) Hepatic fibrosis was assessed by Sirius red staining and quantitatively expressed as the fraction of Sirius red–positive area, which was quantified automatically from whole-slide scans. All figures are representative of experiments conducted from 3 independent samples showing no statistical differences.

To further validate this observation, we assessed the activation of primary murine HSCs (pmHSCs) isolated from p70S6K^–/–^ mice or syngeneic age-matched wild-type (Wt) control mice. While assessing the phenotype of spontaneous ageing of Wt vs p70S6K^–/–^ mice, we did not detect differences in body to liver or spleen weight, or in liver serum biochemistry between the 2 strains (Figure 5*A* and *B*). Also, there were no differences in liver histology and fibrosis markers as judged by Western blot or Sirius red staining (Figure 5*C*–*E*). However, when we examined the ex vivo activation of pmHSCs isolated from p70S6K^–/–^ mice vs Wt mice, we observed a significantly lower increase of α-SMA expression in pmHSCs isolated from p70S6K^–/–^ mice in comparison with Wt mice throughout a 13-day time period (Figure 4*C*). This suggests that p70S6K regulates HSC response to activation by external fibrogenetic stimuli. The findings on p70S6K activation in these 3 model systems pointed to a causal role of p70S6K in determining activation of HSC and α-SMA expression in both human and murine cells.

### p70S6K knockout confers protection from liver fibrosis in mice

**Figure 6:**
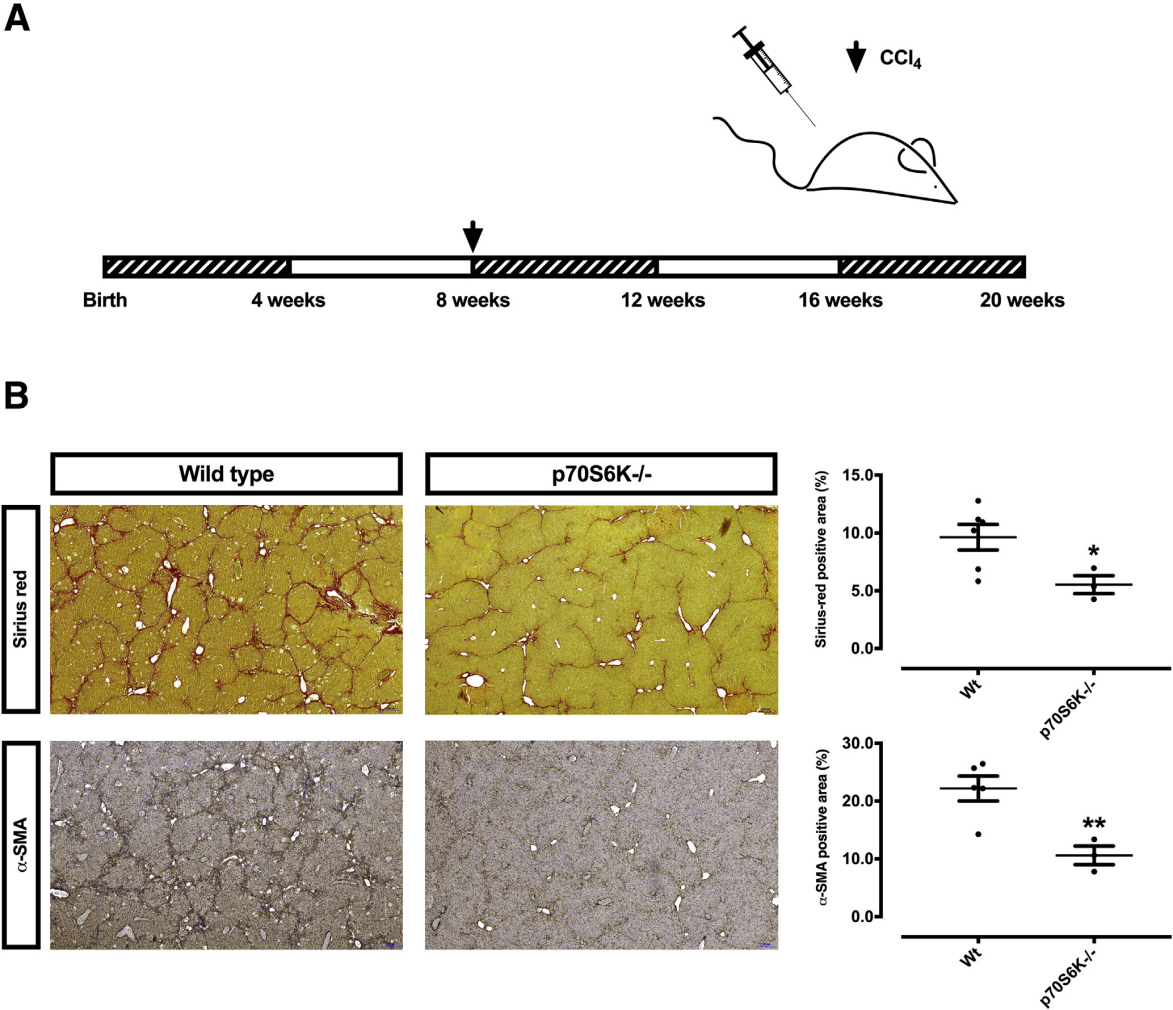
**p70S6K knockout confers protection from liver fibrosis in mice.** (*A*) The time course of in vivo experiments with Wt and p70S6K^–/–^ male mice, which were challenged with CCl_4_ for altogether 12 weeks. (*B*) Hepatic fibrosis was assessed by Sirius red staining and immunohistochemistry for α-SMA. The positive area was quantified automatically from whole-slide scans (n = 3 per group; ∗*P <* .05; ∗∗*P <* .01; *t* test).

**Figure 7:**
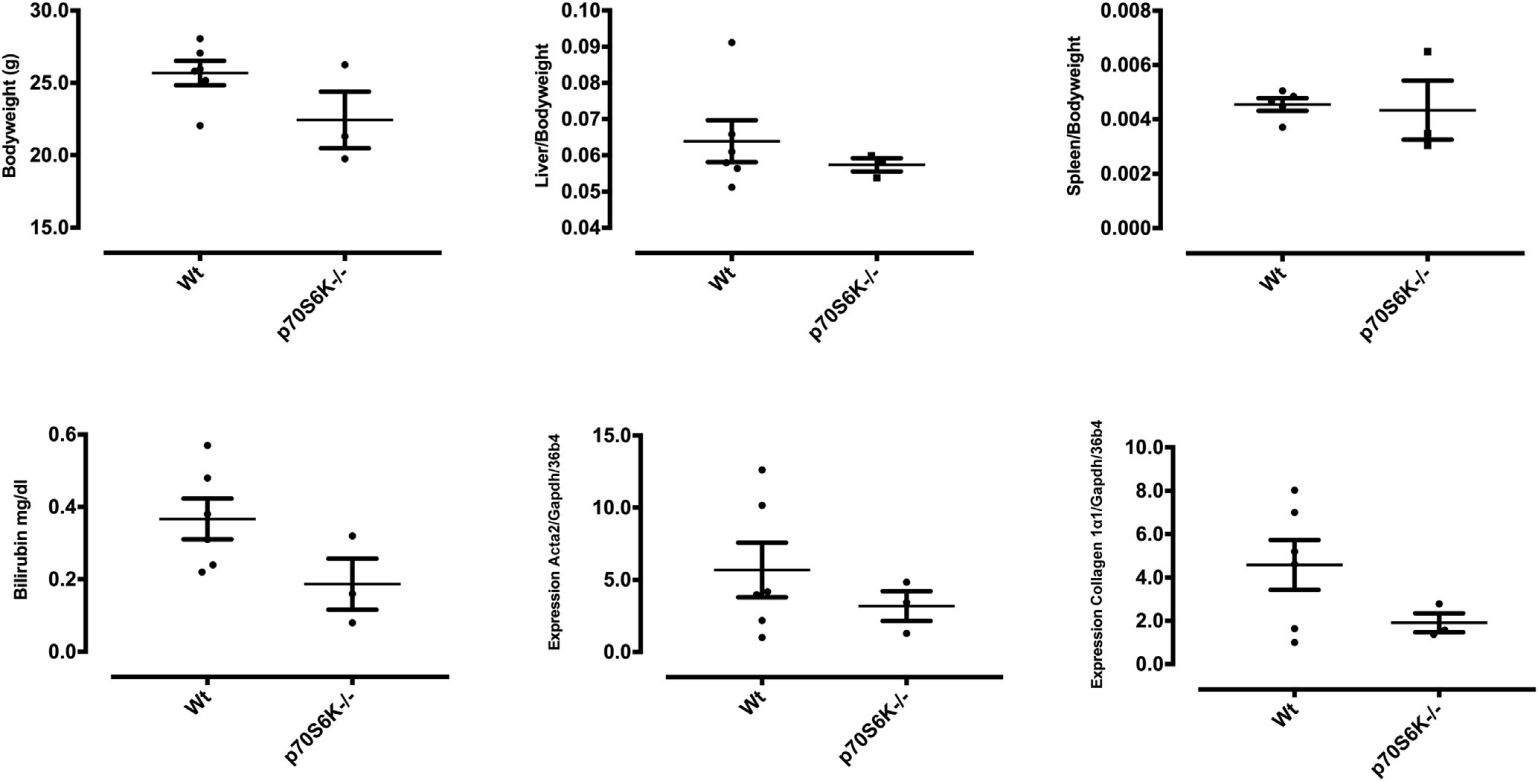
**CCl_4_ challenge in Wt vs p70S6K^–/–^ mice.** (*A*) Male Wt and p70S6K^–/–^ mice were challenged with CCl_4_ for 12 weeks. Body, liver, and spleen weight; bilirubin; and messenger RNA expression of Acta2 and Collagen 1α1 were assessed (n = 3 per group).

To validate these results, an in vivo model was employed, with the hypothesis that a lesser degree of fibrosis should be expected in p70S6K^–/–^ mice upon exposure to chemically induced fibrogenetic stimuli. Male Wt and p70S6K^–/–^ mice were thus challenged for 12 weeks with CCl_4_ (Figure 6*A*). In agreement with the in vitro experiments, we observed that p70S6K^wt^ mice exhibited a much higher extent of fibrosis vs their p70S6K^–/–^ counterpart, as measured by Sirius red and α-SMA staining of liver sections from the respective strains (Figure 6*B*). This was accompanied by numerically lower (though not significant) serum bilirubin values and hepatic Acta2 and collagen 1α1 messenger RNA expression (Figure 7).

### The effects of p70S6K silencing can be reproduced by CEP-1347 in vitro

**Figure 8:**
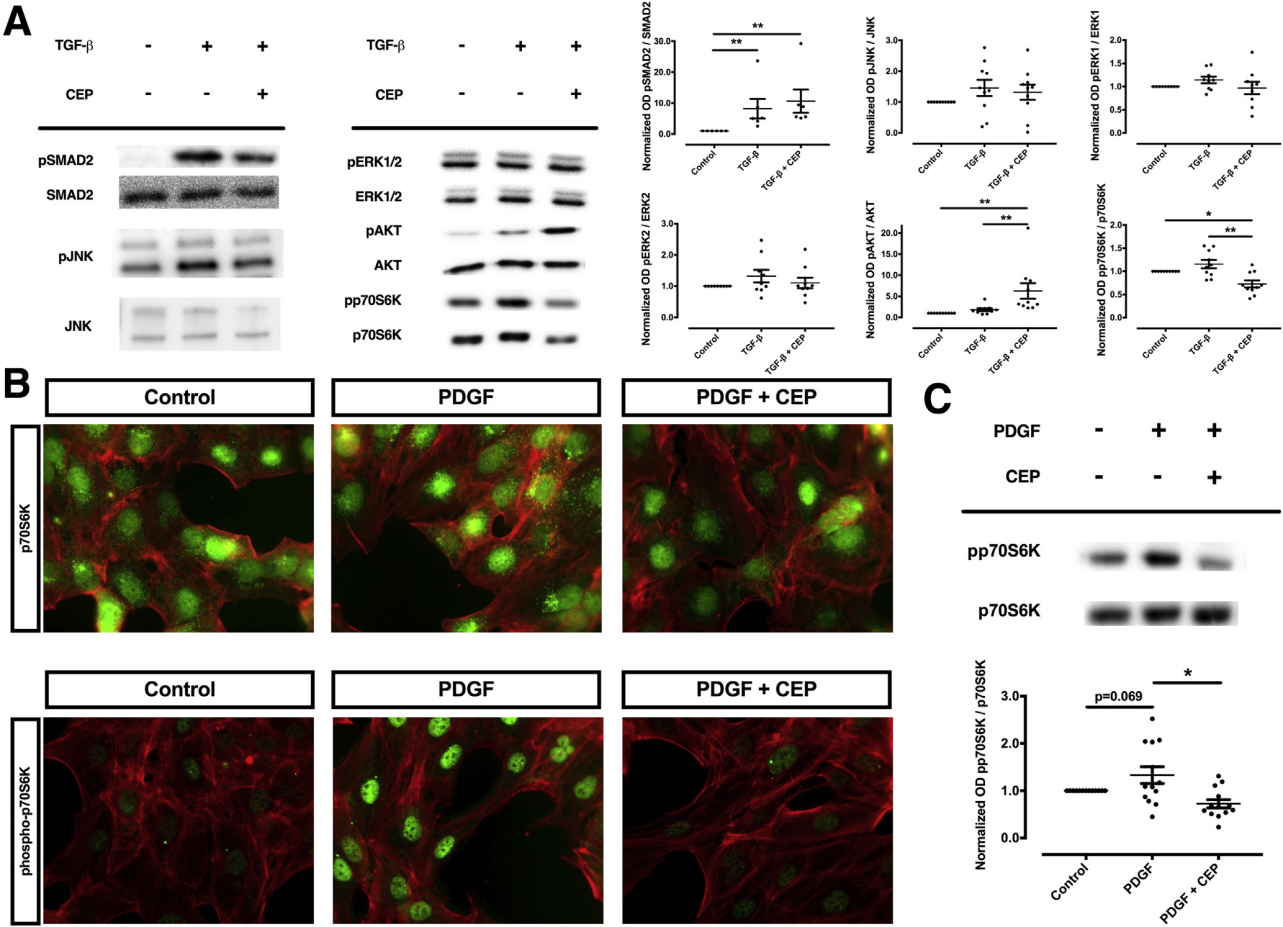
**CEP-1347 does not inhibit phosphorylation of JNK, ERK1, ERK2, or SMAD2 but reduces phosphorylation of p70S6K in human HSCs (LX-2).** Human LX-2 cells were stimulated with (*A*) TGF-β or (*B*, *C*) PDGF-BB and co-stimulated with 1000 nM CEP-1347 (CEP) for (*A*) 24 hours or (*B*, *C*) 1 hour. (*A*) Densitometry data of phosphorylated protein expression levels were normalized to that of unphosphorylated proteins. The results are illustrated as representative blots (at least 6 per group; ∗*P <* .05; ∗∗*P <* .01; analysis of variance). (*B*) Phosphorylation of p70S6K (pp70S6K) was assessed by immunomicroscopy (green) and compared with unphosphorylated p70S6K. (C) Protein expression of pp70S6K was quantified by densitometry. Densitometric values of p70S6K were normalized to unphosphorylated p70S6K (n = 13; ∗ *P* < .05; analysis of variance).

**Figure 9:**
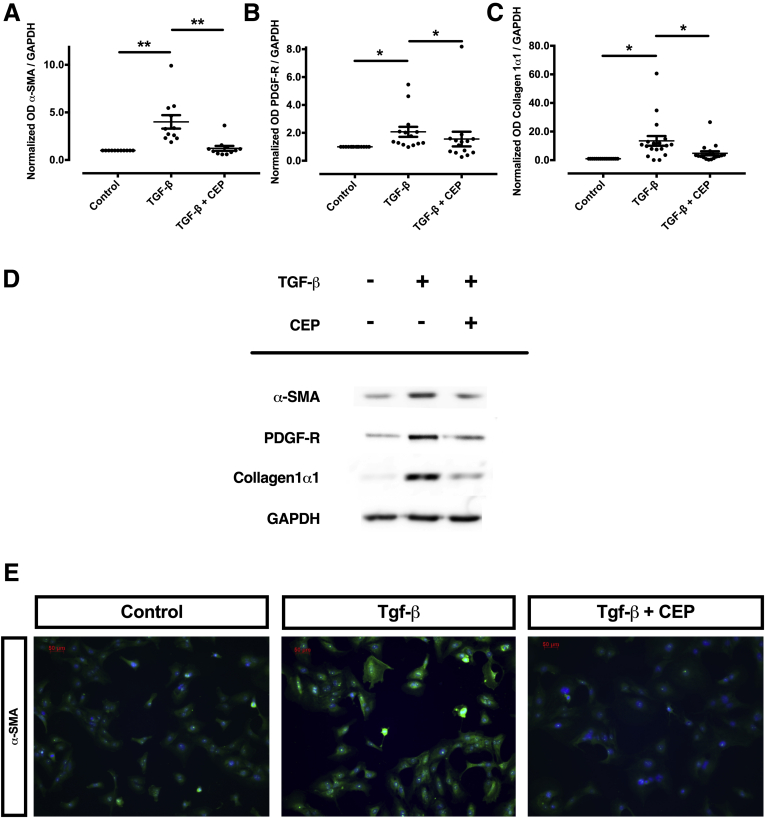
**CEP-1347 inhibits TGF-β–dependent activation of human HSCs (LX-2).** (*A–E*) Human HSCs (LX-2) were activated by TGF-β in the presence or absence of CEP-1347 at the concentration of 1000 nM (CEP) for 24 hours before being harvested for immunoblotting or fluorescence microscopy. (*A*) Representative Western blot of α-SMA, PDGF receptor-β, and collagen 1α1, and (*B–D*) corresponding quantitative optical densitometry are shown. Densitometry data of protein-expression levels from at least 11 replicates were quantified (at least 11 per group; ∗ *P <* .05; ∗∗*P <* .01; analysis of variance). (*E*) Representative fluorescence microscopy pattern of HSCs for α-SMA staining (green). Cell nuclei, counterstained with Hoechst, are visible in blue.

**Figure 10:**
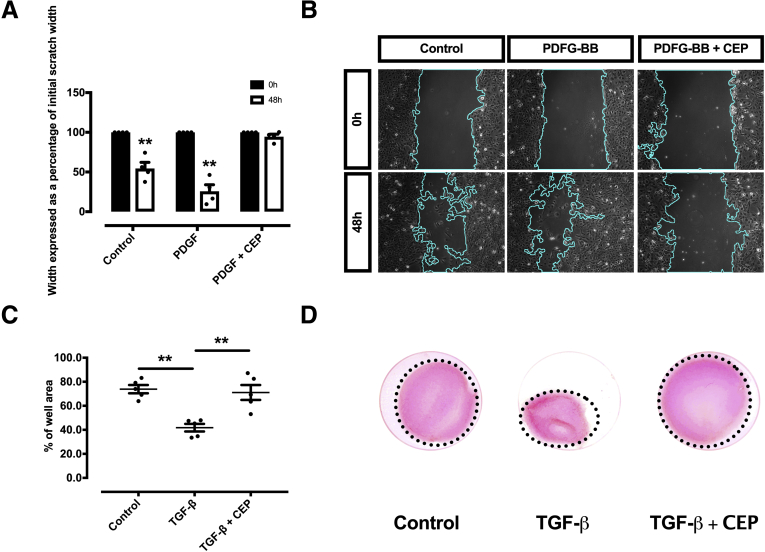
**CEP-1347 inhibits TGF-β–dependent migration and contractility of human HSCs (LX-2).** (*A*, *B*) Representative figures of a migration assay upon stimulation with PDGF-BB and CEP-1347 (CEP). The reduction in gap size was quantified at baseline (left) and at 48 h (right) (n = 4; ∗∗*P <* .01; *t* test). (*C*, *D*) Collagen matrix was populated with human LX-2 cells and contractility induced by addition of TGF-β with or without CEP-1347 for 120 hours. The gel area is expressed as a percentage of the well-area (n = 5; ∗∗*P <* .01; analysis of variance).

In a next set of experiments, we aimed at assessing whether p70S6K can be inhibited pharmacologically. To this purpose, we used CEP-1347, a kinase inhibitor which we established as an effective inhibitor of p70S6K (Figure 8*A*–*C*). When LX-2 cells were incubated with TGF-β in the presence or absence of CEP-1347, the activation of this HSC cell line was almost completely inhibited. Specifically, CEP-1347 strongly inhibited the induction of α-SMA, PDGF receptor-β, and collagen 1α1 protein-expression (Figure 9*A*–*E*) as well as the migratory capacity and the contractility of HSCs (Figure 10*A*–*D*). The capability of CEP-1347 to inhibit p70S6K was demonstrated by a significantly diminished phosphorylation of p70S6K when cells were incubated with TGF-β in the presence of CEP-1347 (Figure 8*A*). Similar results were obtained when PDGF-BB (a well-established inducer of the PI3K-AKT-mTOR signaling in HSCs)^22^^,^^23^ was used to activate HSCs (Figure 8*B* and *C*). In comparison, CEP-1347 did not show any relevant effect on other kinases known to affect fibrogenesis (Figure 8*A*).

**Figure 11:**
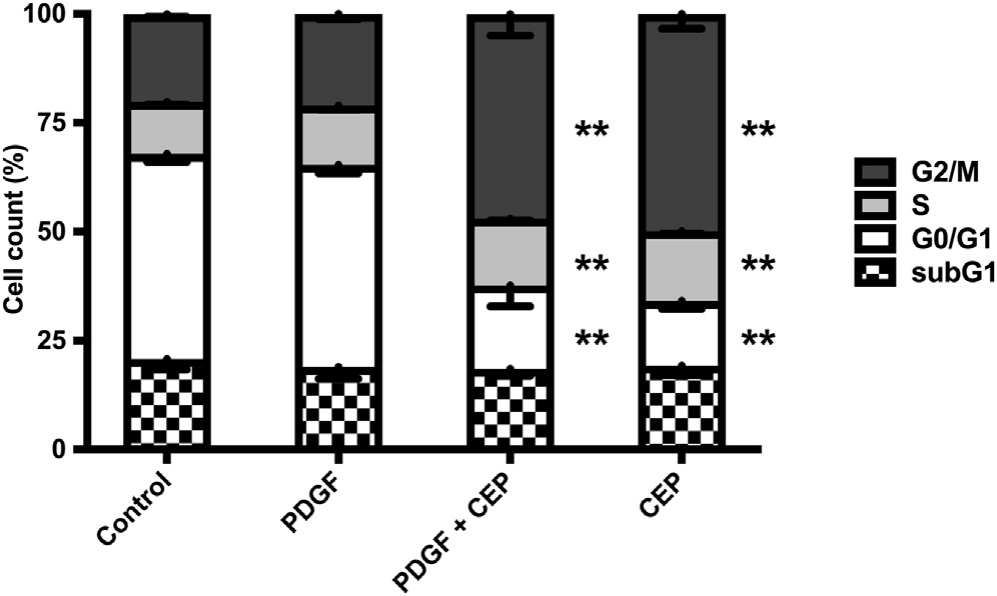
**CEP-1347 induces G2/M cell cycle phase arrest in human HSCs (LX-2).** Cell cycle was evaluated using fluorescence-activated cell sorting analysis after being incubated for 24 hours with PDGF-BB, CEP-1347, or their combination (n = 6; ∗∗*P <* .01; analysis of variance).

**Figure 12:**
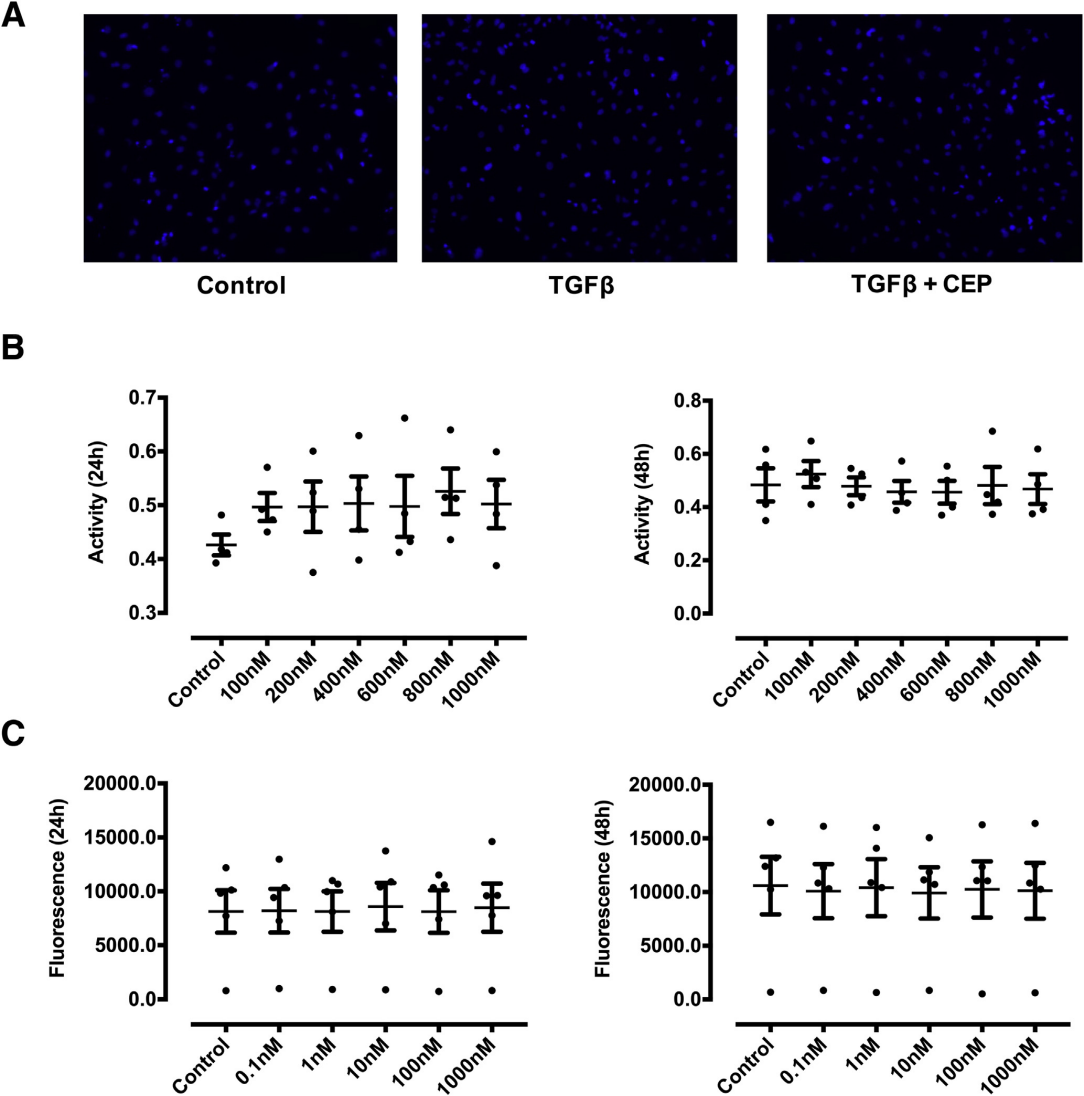
**Effect of CEP-1347 on apoptosis and cell viability in LX-2 cells.** (*A*) The effect of CEP-1347 (CEP) on apoptosis was assessed in LX-2 cells by Hoechst 33342 staining, which failed to show alteration of nuclear chromatin staining upon incubation with CEP-1347 at the concentration of 1000 nM vs control cells. (*B*, *C*) No decrease of cell viability was detected by using water-soluble tetrazolium assays and Pico-Green 24 and 48 hours after stimulation with the indicated concentrations of CEP-1347 (data are representative of measurement from at least 4 different experiments).

**Figure 13:**
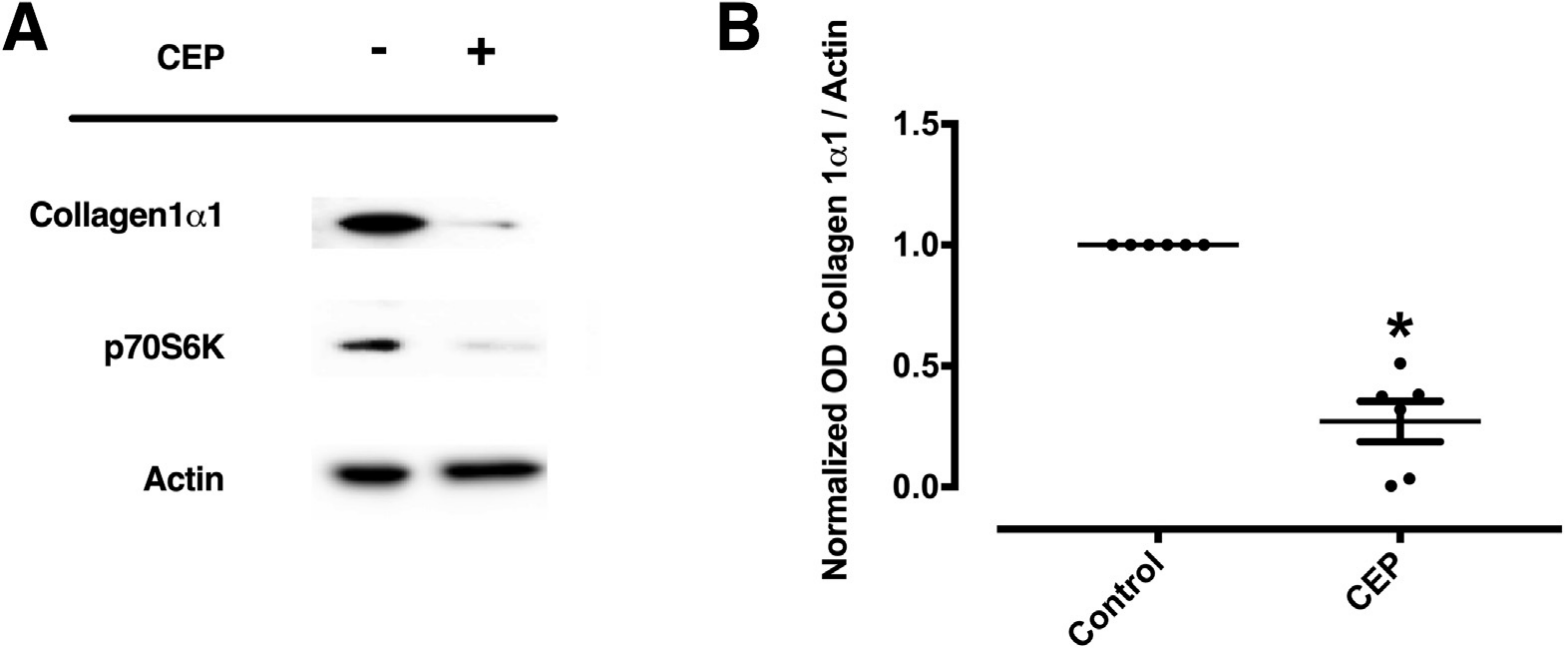
**CEP-1347 inhibits p70S6K protein expression and collagen 1α1 deposition in phHSCs.** PhHSCs were cultured on uncovered plastic to induce spontaneously activation for 7 days. Fresh media with 1000nM (CEP) were replaced 3 times a week. (*A*, *B*) Expression of collagen 1α1 and p70S6K were determined by Western blot and quantified by densitometry (n = 7; ∗*P <* .05; Wilcoxon test).

Interestingly, CEP-1347 induced a significant G2 cell cycle arrest (Figure 11), an effect that might contribute to decreased fibrogenesis of HSCs. However, the possibility that this occurred solely as a consequence of a loss of cell viability caused by CEP-1347, rather than as a consequence of a direct antifibrotic effect of this drug, was excluded by viability assays, Hoechst staining and fluorescence-activated cell sorting analysis, which failed to show a relevant decrease in cell viability or apoptosis within the time frame in which antifibrotic effects were observed (Figures 11 and 12). These results were further validated in experiments with phHSCs, showing that CEP-1347 inhibits p70S6K along with collagen 1α1 expression (Figure 13*A* and *B*). These results show that CEP-1347 effectively inhibits p70S6K, hereby counteracting the profibrotic effects of HSC activation in different models.

## Discussion

### Progression of liver disease to fibrosis and cirrhosis as unmet medical challenge

Many advances have been made in the prevention and causal treatment of CLD, including the widespread use of anti-Hepatitis B virus vaccines and antiviral drugs, with the latest and most significant achievement being the development of direct-acting antiviral agents for the treatment of chronic hepatitis C.^24^ For other CLDs, however, causal treatment is still lacking or poorly effective. Until individual curative etiological treatment options are established for all etiologies of CLD, the development of direct-acting agents capable of preventing progression of CLD to liver cirrhosis and its complications represents an important strategy to reduce CLD-related mortality. The importance of this approach is exemplified by NASH, a condition poorly responsive to treatment and due to become the leading cause of CLD globally.^25^^,^^26^ However, attempts at establishing clinically successful primary antifibrotic agents have so far failed.^11^, ^12^, ^13^ While setting out to investigate alternative molecular targets for antifibrogenic treatment, we chose to focus on p70S6K, as this molecule is located downstream of the PI3K-AKT-mTOR signaling, where several pathways of fibrogenesis converge. We speculated that p70S6K might be a potential downstream effector of fibrogenesis. Thus, we expect that the effects of its pharmacological inhibition should be restricted to the prevention of collagen deposition, limiting the possible spectrum of adverse effects common to upstream regulators of this axis. Three topics describe the main findings of our article and their clinical significance.

### P70S6K is a central mediator of fibrogenesis downstream of PI3K-AKT-mTOR signaling

Firstly, we provide novel mechanistic data on fibrogenesis by describing p70S6K as a distal effector of fibrosis downstream of mTOR activation by consistent results obtained in cell lines, isolated primary cells, human tissues, and p70S6K^–/–^ mice. The several-fold increase of in p70S6K staining observed in cirrhotic human tissue suggested that p70S6K plays an important role in fibrogenesis, an indication supported by the fact that p70S6K also progressively increased during the process of activation of isolated phHSCs in vitro. The causal role of p70S6K in inducing liver fibrosis was confirmed by experiments showing that (1) silencing of p70S6K abolishes activation in human HSCs (Figure 4*A* and *B*); (2) p70S6K^–/–^ HSCs are protected from fibrogenic activation (Figure 4*C*); (3) the effects of p70S6K silencing could be reproduced by CEP-1347, a clinically viable drug that we established as an effective inhibitor of p70S6K (Figure 8*A–C*); and (4) when exposed to CCl_4_, p70S6K^–/–^ mice exhibited decreased liver fibrosis compared with Wt mice (Figure 6). These results are in line with the body of evidence that established the importance of the PI3K-AKT-mTOR axis in determining liver fibrosis and with 2 previous reports assessing the role of p70S6K in a rodent NASH model^27^ and in bile acid–induced activation of HSCs.^28^ We are aware of the limitation of our study that we cannot totally rule out that p70S6K expressed in other cell types (including F4/80 and CK19-positive cells) might also contribute to fibrogenesis.

Staining of p70S6K was not limited to HSCs. Future studies using cell-specific knockout models may shed more light in the role of p70S6K for hepatic fibrogenesis in specific cell compartments. Nevertheless, the current knowledge on the role of HSCs in fibrogenesis supports a likely function of p70S6K as a checkpoint of liver fibrogenesis by HSC activation.

### Clinical implications

However, our results are not only relevant in terms of the definition of the mechanistic role of p70S6K within the pathways regulating fibrogenesis; our finding defining CEP-1347 as an effective inhibitor of this molecule has important potential clinical implications. Owing to the role of PI3K-AKT-mTOR signaling in the regulation of immune response and cell proliferation, several inhibitors of PI3K (comprising copanlisib, duvelisib, or idelalisib) and mTOR (sirolimus, everolimus) have been approved as anticancer or immunosuppressive agents.^29^^,^^30^ However, the use of these substances to prevent the progression of liver fibrosis does not seem to be feasible in the setting of long-term treatment required to treat CLD, owing to potential adverse events anticipated by the well-known antiproliferative or immunosuppressive effects of these agents. Targeting p70S6K would restrict the blockage of PI3K-AKT-mTOR axis to its effect on liver fibrosis without interfering with the actions of the upstream members of this signaling pathway, hereby representing a potentially safer and more tolerable therapeutic strategy. However, to our knowledge, no clinically viable p70S6K inhibitor had been previously described.

### Clinical use of CEP-1347

Here, we suggest for the first time the kinase inhibitor CEP-1347 as an effective inhibitor of p70S6K. CEP-1347 was developed to treat Parkinson’s disease and has proved to be safe, well tolerated, and devoid of long-term unwanted effects in a large cohort of patients (cumulatively, 1467 patient-years).^20^^,^^31^ The extensive clinical data already available and the excellent safety profile of CEP-1347 fulfils a stringent prerequisite for its clinical assessment as a therapeutic agent for CLD and liver fibrosis, alone or in combination with established causal treatment of CLDs.

## Conclusion

In summary, we describe p70S6K as a checkpoint of fibrogenesis and as an actionable target for CEP-1347, a clinically viable and available compound that can safely be administered for long-term treatment. Further studies should be conducted to investigate p70S6K as a therapeutic target to treat CLD.

## Materials And Methods

### Cell lines

LX-2 cells were purchased from Merck Millipore (Darmstadt, Germany). Their authenticity was assessed by the Leibniz-Institut (DSMZ-Deutsche Sammlung von Mikroorganismen und Zellkulturen GmbH), and confirmed based on DNA-fingerprinting.

### Human samples

The use of all human material presented in this article was approved by the ethics committee of the Faculty of Medicine of the University of Munich (Project ID: 17-619). The material was provided by the Biobank under the administration of the Human Tissue and Cell Research Foundation at the Hospital of the University of Munich. The framework of the Human Tissue and Cell Research Foundation (http://www.htcr.org)^32^ includes written informed consent from all donors, and had been previously approved by the ethics committee of our institution (approval Nr. 025-12) as well as by the Bavarian State Medical Association (approval Nr. 11142). The biobank and the cell isolation core facility of the department of surgery have implemented total quality management and are certified to ISO 9001:2008. The study protocol conforms to the ethical guidelines of the 1975 Declaration of Helsinki as reflected in a priori approval by the institution's human research committee.

### Cell culture

Cells were kept in Iscove Basal Medium (Merck Millipore; phHSCs) or in Dulbecco’s modified Eagle medium (Sigma-Aldrich, Darmstadt, Germany; pmHSCs and LX-2 cells) containing 2% (LX-2) or 10% (phHSCs and pmHSCs) fetal bovine serum (Merck Millipore) and antibiotics (Sigma-Aldrich). All cells were cultivated in a humidified atmosphere with 5% CO_2_ and 21% O_2_ at 37°C. LX-2 cells were stimulated with 10 ng/mL TGF-β (PeproTech, Hamburg, Germany) or 30 ng/mL PDGF-BB (Biomol, Hamburg, Germany) and co-incubated with 1000 nM CEP-1347 (Tocris, Bristol, United Kingdom) for 1 hour, 24 hours, or 48 hours, where indicated. phHSCs and pmHSCs were cultured on uncovered plastic dishes for up to 13 days to induce a spontaneous activation. For these long-term experiments, culture media in phHSCs were replaced with fresh media and CEP-1347 3 times a week.

### Isolation of phHSCs

**Figure 14:**
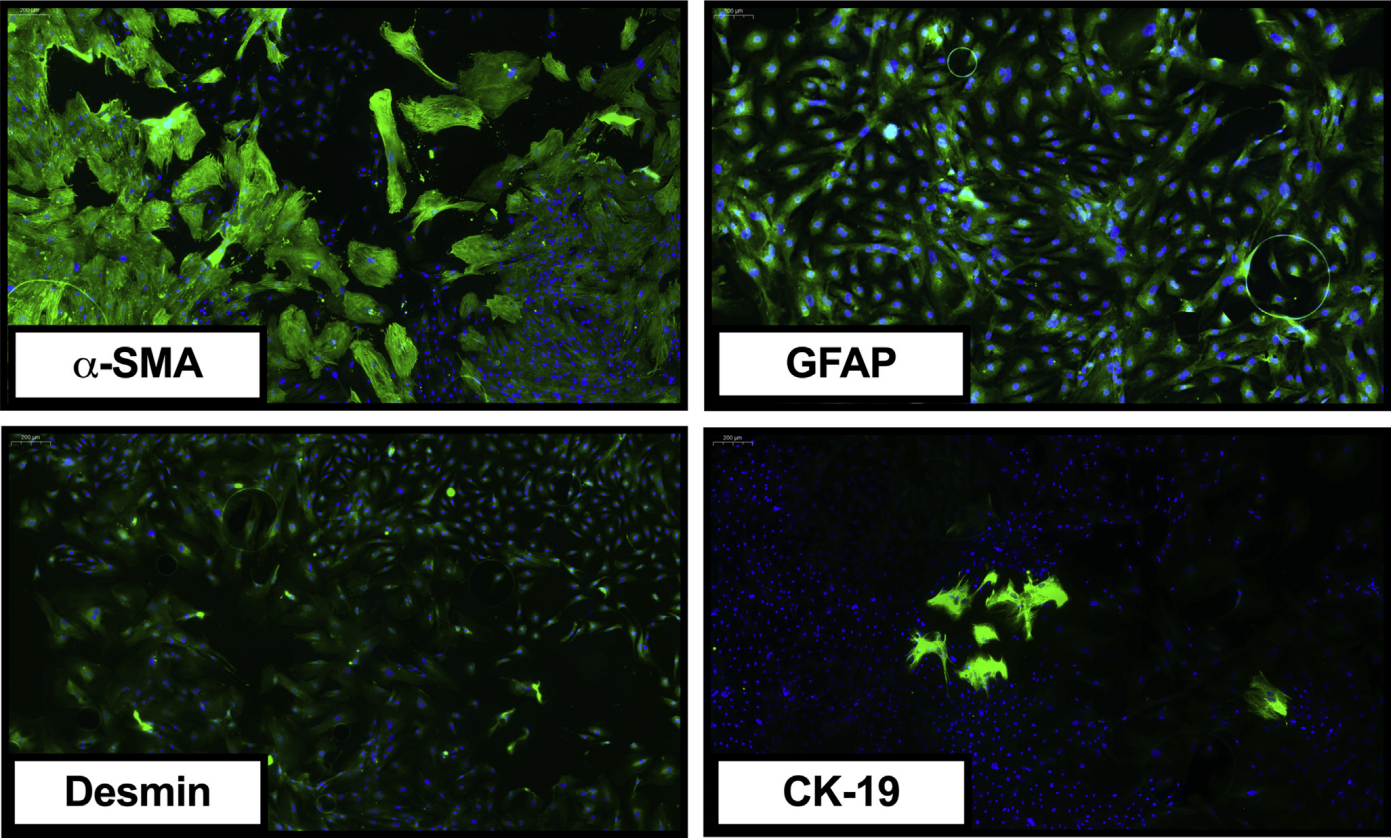
**Characterization of isolated phHSCs.** Representative immunocytomicroscopy figures showing that more than 95% of isolated cells exhibit typical markers of HSCs, including α-SMA, GFAP, and Desmin.

A liver cell suspension was prepared using a 2-step collagenase perfusion technique with modifications as described previously.^33^^,^^34^ In brief, hepatocytes were removed by multiple cycles of low-speed centrifugation, which resulted in a supernatant that contained the nonparenchymal cells. phHSCs were subsequently purified from the nonparenchymal cell fraction by discontinuous density centrifugation using Percoll.^33^ Quality of isolation was checked using immunocytomicrocopy against α-SMA (Sigma-Aldrich), Desmin (Sigma-Aldrich), GFAP (Sigma-Aldrich), and CK-19 (Merck Millipore) (Figure 14).

### Isolation of pmHSCs

Isolation of pmHSCs was performed by pronase-collagenase perfusion followed by density gradient centrifugation in 13.2% Nycodenz (Axis-Shield PoC, Oslo, Norway) as described previously.^21^

### Viability assays

The effect of CEP-1347 on cell viability was assessed by 2 kinds of viability assays—PicoGreen (Invitrogen, Waltham, MA) and by water-soluble tetrazolium assay (Promega, Walldorf, Germany)—according to the instructions provided by the manufacturers.

### Western blot

Proteins were loaded in equal amounts and separated by sodium dodecyl sulfate polyacrylamide gel electrophoresis and transferred onto polyvinylidene difluoride membranes (Merck Millipore). Membranes were incubated with the primary antibodies directed against the following molecules: α-SMA (Sigma-Aldrich) PDGF receptor-β (Cell Signaling Technology, Danvers, MA), collagen 1α1 (R&D Systems, Minneapolis, MN), JNK, phospho-JNK (pJNK), ERK1/2, phospho-ERK1/2 (pERK), SMAD2, phospho-SMAD2 (pSMAD2), AKT, phospho-AKT (pAKT), p70S6K, phospho-p70S6K (pp70S6K) (all from Cell Signaling Technology), GAPDH (Abcam, Cambridge, United Kingdom) and β-actin (Sigma-Aldrich). The following secondary antibodies were used as needed: a goat-anti-mouse-IgG-HRP antibody (Bio-Rad, Feldkirchen, Germany), a goat-anti-rabbit-IgG-HRP (Bio-Rad), or a goat-anti-sheep-IgG-HRP antibody (R&D Systems). Visualization was performed using the ChemoCam (INTAS, Homburg, Germany) after incubation with Clarity Western ECL Substrate (Bio-Rad).

### Real-time polymerase chain reaction

Real-time polymerase chain reaction was performed in a SYBR Green system (QuantiTect SYBR Green PCR Kit; Qiagen, Venlo, the Netherlands) using a LightCycler 96 (Roche, Penzberg, Germany). Expression was calculated according to the ΔΔCt method with Gapdh and 36b4 as the housekeeping genes and normalized to the means of the controls. The following primer sequences were used:

Gapdh: Fwd.: AGTATGACTCCACTCACGGC Rev.: ATGTTAGTGGGGTCTCGCTC

36b4: Fwd.: TCTAGGACCCGAGAAGACCT Rev.: CCCACCTTGTCTCCAGTCTT

Acta2: Fwd.: GAGACTCTCTTCCAGCCATCTT Rev.: CCCTGACAGGACGTTGTTAGC

Collagen 1α1: Fwd.: CCTGGCAAACAAGGTCCTTC Rev.: GGATCCCTCACGTCCAGATT.

### Hoechst staining and immunocytomicroscopy

Cells were stimulated for 24 hours with 1000 nM CEP-1347 before undergoing incubation with Hoechst 33342 (Fluka, Buchs, Switzerland). For immunocytomicroscopy following antibodies were used: α-SMA (Sigma-Aldrich), p70S6K (Cell Signaling Technology), phospho-p70S6K (Cell Signaling Technology), CK-19 (Merck Millipore), Desmin (Sigma-Aldrich), and GFAP (Sigma-Aldrich). As secondary antibody, an Alexa-Fluor 488 goat anti-mouse IgG or goat anti-rabbit IgG was used. Hoechst or phalloidin (Invitrogen) were used for counterstaining. Microscopy was performed using a Zeiss Axiovert TV 135 (Zeiss, Oberkochen, Germany).

### Immunohistochemistry and immunofluorescence

Paraffin-embedded sections (3 μm) of primary human or primary murine liver tissues were used for p70S6K or α-SMA immunohistochemical staining. p70S6K monoclonal rabbit antibody (Cell Signaling Technology) or α-SMA polyclonal rabbit antibody (Abcam) were applied as primary antibodies and detected by EnVision+System-HRP–labeled polymer anti-rabbit (Dako, Santa Clara, CA). For immunofluorescence studies, human or murine liver tissues were co-incubated with p70S6K monoclonal rabbit antibody (Cell Signaling Technology) and anti-α-SMA monoclonal mouse antibody (Sigma-Aldrich) GFAP mouse antibody (Sigma-Aldrich), F4/80 monoclonal mouse antibody (Santa Cruz Biotechnology, Dallas, TX), CK19 monoclonal mouse antibody (Proteintech, Rosemont, IL), or monoclonal mouse antibody in 5% bovine serum albumin (Merck Millipore), and detected with Alexa Fluor 488 goat anti-rabbit IgG (Invitrogen, Darmstadt, Germany) and Alexa Fluor 594 goat anti-mouse IgG (Invitrogen). Nuclei were counterstained with Vectashield (Vector Laboratories, Burlingame, CA) containing Hoechst 33342 (Sigma-Aldrich). Pictures were taken using the Leica fluorescence microscope (Leica Microsystems, Wetzlar, Germany).

### Sirius red staining

Liver samples were fixed using 4% formaldehyde. After embedding in paraffin, 4-μm sections were stained with Sirius red according to standard protocols.

### Quantification of positive stained area

Slides from human or murine liver tissue were scanned as indicated after Sirius red or after immunhistochemical staining of p70S6K, α-SMA, and collagen 1α1 using a Pannoramic MIDI II digital slide scanner from 3D-Histech (Sysmex, Norderstedt, Germany). The stained fibrotic area was quantified via QuPath software (https://qupath.github.io/) and ImageJ2 software (National Institutes of Health, Bethesda, MD) as described previously.^35^

### Si-RNA silencing

p70S6K siRNA was purchased from Qiagen; β-galactosidase siRNA, which was used as a control, was purchased from Dharmacon (Lafayette, CO). Lipofectamin 2000 (Invitrogen) was used as transfectant for siRNA experiments. LX-2 cells were treated 48 hours after transfection with 10 ng/mL TGF-β for 24 hours. For siRNA transfection in phHSCs, cells were cultured on uncovered plastic for 7 days. Transfection with the respective p70S6K and control siRNA was performed on day 2 and day 5.

### Fluorescence-activated cell sorting

For analysis of cell cycle, cells were stimulated with PDGF-BB, CEP-1347 or their combination for 24 hours before being harvested and undergoing propidium iodide staining (Sigma, Germany) as described previously. Fluorescence-activated cell sorting analysis was performed by using the Accuri C6 flow cytometer (BD Biosciences, Franklin Lakes, NJ) and its built-in software.

### Gel-contraction assay

The gel-contraction assay was performed according to the method described by Ikenaga et al.^36^ After polymerization of the collagen, the gel was mobilized from the surface using a pipette tip. Medium containing stimulating agents was added to the gels. After 24 hours of incubation, the gel area was measured using ImageJ software. The ratio of the well area to the gel area was calculated.

### Migration assay

LX-2 cells were seeded in 6-well plates. When cells reached confluence, a horizontal scratch was made with the tip of a 100-μL pipette. Afterward, the wells were washed once with cell culture medium and stimulated with the different agents for 24 hours. Images were acquired before and after incubation with these agents to determine (ImageJ software) the reduction of the initial cell-free area as marker of cell migration activity.

### Animal experiments

Liver fibrosis was induced using CCl_4_ (Sigma-Aldrich) dissolved in corn oil (Sigma-Aldrich) as described previously.^35^ Male C57BL/6 p70S6K^+/+^ (Wt) vs p70S6K^–/–^ mice were bred from p70S6K^+/-^ mice (purchased from RIKEN [Tokyo, Japan]) and challenged 3 times weekly with intraperitoneal injections of CCl_4_. A first dose of 0.25-mL CCl_4_ per kg body weight was administered when animals reached the eighth week of age and was followed by injections of 0.5-mL CCl_4_ per kg body weight for altogether 3 months.^35^ Animals were kept on a 12-hour light/dark cycle, fed ad libitum, and received human care in compliance to the standard local and international regulations outlined in the Guide for the Care and Use of Laboratory Animals. All experiments were approved by the local authorities (Regierung von Oberbayern). All experiments conforms to the ARRIVE guidelines.

### Figures

Figures were created using GraphPad Prism 7 (GraphPad Software, San Diego, CA) or Adobe Illustrator CC 2019 (Adobe, San Jose, California).

### Statistical analysis

Statistical calculations were performed by using SPSS 25 (IBM, Armonk, NY) or GraphPad Prism 7 using analysis of variance, *t* test, Mann-Whitney *U* test, or Wilcoxon or Kruskal-Wallis test as indicated in the presentation of the specific experiments. When a relevant influence of an experiment was observed, univariate analysis of variance was performed and degrees of freedom, means of squares, and *F* values were calculated. *P* values lower than .05 were referred to as statistically significant. All data are presented as mean ± SEM.
